# Beneficial Effect of *Gastrodia elata* Blume and *Poria cocos* Wolf Administration on Acute UVB Irradiation by Alleviating Inflammation through Promoting the Gut-Skin Axis

**DOI:** 10.3390/ijms231810833

**Published:** 2022-09-16

**Authors:** Ting Zhang, Shaokai Huang, Jingyi Qiu, Xuangao Wu, Heng Yuan, Sunmin Park

**Affiliations:** 1 Department of Bioconvergence System, Hoseo University, Asan 31499, Korea; 2 Department of Food and Nutrition, Obesity/Diabetes Research Center, Hoseo University, Asan 31499, Korea

**Keywords:** UVB, *Gastrodia elata* Blume, *Poria cocos*, photoaging, metagenome analysis

## Abstract

Bioactive compounds in some herbs can, directly and indirectly, protect against photoaging. We evaluated the effects of *Gastrodia elata* Blume (GE) and *Poria cocos* Wolf (PC) water extracts on ultraviolet (UV) B-induced skin lesions by acute UVB exposure in ICR mice and explored their mechanism of action. After removing the hair on the back of the mice, UVB (280–310 nm) was exposed to the back for 30 min to induce skin damage. Four UVB exposure groups were divided into the following according to the local application (1,3-butanediol extract) on the dorsal skin and oral intake (0.3 g water extract/kg body weight/day): 1,3-butanediol and cellulose(control; UV-Con), retinoic acid (positive-control; UV-Positive), PC extracts (UV-PC), and GE extracts (UV-GE). The fifth group had no UVB exposure with the same treatment as the UV-Con (Normal-control). The erythema, burns, erosion, and wounds of the UV-PC and UV-PC groups were alleviated, and the most significant improvements occurred in the UV-PC group. PC and GE reduced the thickness of the dorsal skin tissue, the penetration of mast cells, and malondialdehyde contents. The mRNA expression of *TNF-α*, *IL-13*, and *IL-4*, inflammatory factors, were also reduced significantly in the dorsal skin of the UV-PC and UV-GE groups. UV-PC, UV-GE, and UV-Positive showed improvements in UV-induced intestinal tissue inflammation. UV-Con deteriorated the intestinal morphology, and PC and GE alleviated it. The α-diversity of the fecal microbiota decreased in the UV-control, and UV-PC and UV-GE prevented the decrease. Fecal metagenome analysis revealed increased propionate biosynthesis in the UV-PC group but decreased lipopolysaccharide biosynthesis in the UV-PC and UV-GE groups compared to UV-Con. In conclusion, the local application and intake of PC and GE had significant therapeutic effects on acute UV-induced skin damage by reducing oxidative stress and proinflammatory cytokines, potentially promoting the gut-microbiota-gut-skin axis.

## 1. Introduction

Acute intense ultraviolet (UV) B exposure induces sunburn, potentially leading to skin cancer. Sunburn by UVB radiation produces reactive oxygen species (ROS) and proinflammatory cytokines to damage cell DNA and extracellular matrix components [1,2]. In detail, UVB irradiation stimulates the interaction of intracellular chromophores with oxygen molecules to produce ROS that causes direct cell damage, especially DNA, and interacts with proinflammatory cytokine production and immunity [1]. The changes in the DNA in the skin potentially result in skin cancer [3]. Furthermore, skin inflammation is linked to not only skin tissues but also body metabolism and, potentially, gut microbiota.

Gut microbiota is linked to most organs in the body to modulate their functions, primarily through modulating immunity [4]. Skin health is also associated with gut microbiota bidirectionally, and gut microbiota dysbiosis is involved in skin diseases such as atopic dermatitis, psoriasis, acne vulgaris, and skin cancer [5]. Dietary choices such as dietary fiber and polyphenol, lifestyles, and host genetics contribute to gut microbiota [6]. The intestinal microbiota community plays a critical role in the host’s immunity, inflammation, and oxidative stress, contributing to skin health [7]. Herbal extracts contain soluble dietary fiber and polyphenols, and intestinal bacteria alter them as secondary metabolites that the host can absorb and utilize. The intestinal microbiota can reduce the increased ROS and proinflammatory cytokines by UVB exposure, and the secondary metabolites generated by intestinal bacteria attenuate skin damage, called the gut-skin axis [7].

Antioxidants, such as vitamins (vitamin C and vitamin E), flavonoids, and polyphenolic compounds, play a prominent role in fighting against ROS to protect against UVB-induced skin damage, including sunburn [7,8]. Traditional Chinese medicine, containing antioxidants and anti-inflammatory components, is often considered safe and effective for UVB-induced skin damage treatment in China, South Korea, and Japan [9,10]. Various Chinese herbal medicines, tablets, and ointments have been continuously studied and developed as functional foods and cosmetics [11,12]. Furthermore, herbs and herbal preparations have a high potential for UVB-induced skin damage protection and treatment because of their antioxidant and anti-inflammatory activity [12,13].

*Gastrodia elata* Blume (GE), a saprophytic, perennial herb in the Orchidaceae family, is native to several Asian countries, including China, Korea, and Japan. The dry tuber of GE (Gastrodiae rhizome; “Tianma” in Chinese) has been used for centuries in traditional Chinese medicine to treat dizziness, paralysis, and epilepsy [14]. GE has anti-inflammatory and anti-oxidative stress properties and promotes insulin signaling [15]. *Poria cocos* Wolf (*Wolfiporia extensa* Ginns; PC), an edible and pharmaceutical mushroom, has a long history of medicinal use in Asian countries [16]. Various studies of PC have demonstrated its marked anti-inflammatory activity in different experimental animal models of acute and chronic inflammation [17]. Therefore, this study established the hypothesis that the local application and intake of GE and PC extracts attenuated UV-induced skin damage by modulating oxidative stress, inflammation, and gut microbiota. The hypothesis was tested in the UVB-irradiated animal model, known to develop clinical symptoms similar to human UV-induced skin damage after topical exposure to UV light [18]. This study demonstrated that UV-induced skin damage could be alleviated by reducing oxidative stress and inflammation via the gut microbiota-gut-skin axis.

## 2. Results

### 2.1. Total Polyphenol and Flavonoids Contents

GE contained total polyphenol 78.6 ± 0.34 mg GAE/g extract and total flavonoid 32.7 ± 0.41 mg QE/g extract while PC included total phenol 38.5 ± 0.32 mg GAE/g extract and total flavonoid 18.4 ± 0.44 mg QE/g extract.

### 2.2. Energy Metabolism

The final weight and weight gain were lowered in the UV-Con group (UVB irradiation + no herbal treatment) than in the Normal-Con group (no UVB irradiation + no herbal treatment), but kept constant in the UV-PC (UVB irradiation + PC treatment) and UV-Con groups. The UV-GE group (UVB irradiation + GE treatment) showed protection against a decrease, but the UV-Positive group (UVB irradiation + retinoic acid) showed the most significant decrease (Table 1). On the other hand, the food intake was similar among the groups. The food efficiency was lower in the UV-Con group (0.73 ± 0.27) than in the Normal-Con group (1.20 ± 0.27); it was similar in the UV-GE (1.13 ± 0.24) and Normal Con groups and lowest in the UV-Positive group (−0.29 ± 0.21; *p* < 0.05) (Table 1). At the experimental treatment, mice in the UV-Positive group had the lowest visceral fat weight, summing of epididymal and retroperitoneal fat mass. In contrast, the other groups except UV-Positive showed a similar visceral fat mass (*p* < 0.05) (Table 1).

### 2.3. Evaluation of Changes in Skin Lesions in Mice

**Figure 1:**
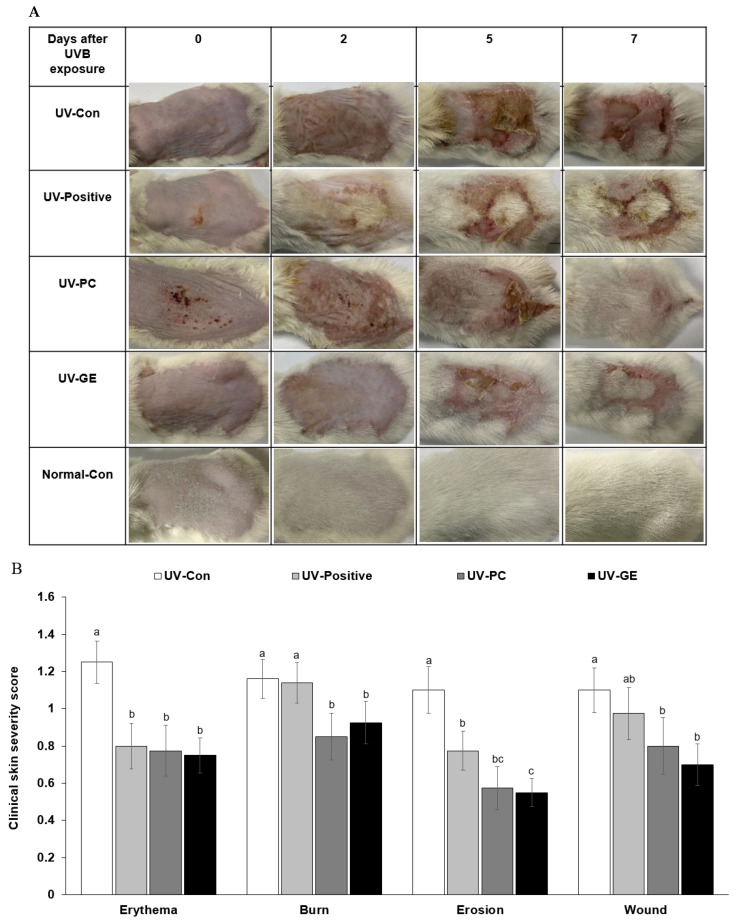

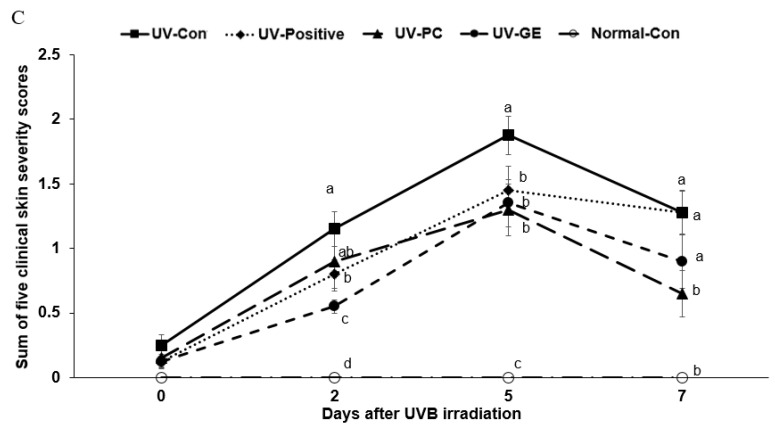
The severity of clinical photoaging symptoms. (**A**) Changes in photoaging symptoms at the dorsal skin lesion from day 14 to day 21. (**B**) The severity of each clinical photoaging symptom on the 21st day. (**C**) Average scores of clinical symptoms by identifying photoaging from day 0 to 7 days. ^a,b,c^^,d^ Means with different superscripts were significantly different among the groups in each parameter by the Tukey test at *p* < 0.05.

The clinical changes by UVB irradiation were evaluated at zero, two, five, and seven days after the UVB exposure to the dorsal skin (Figure 1A). The clinical symptoms included wounds, burns, erythema, erosion, epidermal exfoliation, and skin thickness. In the Normal-Con, the hair on the back skin grew back without clinical symptoms within seven days in Figure 1A. However, the UV-Con mice showed an apparent lesion on the back from day 2 until day 7. However, UV-PC and UV-GE alleviated the lesions on day 7, compared to the UV-Con (Figure 1B). Obvious clinical symptoms appeared in all UV groups on day 2, and from the fifth day onwards, the surface erythema began to decrease in the UV-GE, UV-PC, and UV-Positive groups but not in the UV-Con group (*p* < 0.05; Figure 1B,C). The UV-PC and UV-GE groups showed reduced erosion, burn, erythema, and wound compared to the UV-Con group, and the UV-PC group showed better alleviation compared to the UV-GE group (*p* < 0.05; Figure 1B,C). The total clinical severity scores were higher in the UV-Con group (1.15 ± 0.13) compared to that in the UV-Positive (0.80 ± 0.11) and UV-GE (0.55 ± 0.18) groups on day 2, and they were lower in the UV-GE (1.35 ± 0.18), UV-PC (1.29 ± 0.20), and UV-Positive (1.45 ± 0.17) groups compared to that in UV-Con (1.88 ± 0.15) on day 5. On day 7, the scores were lower in the UV-GE (1.28 ± 0.17) and UV-PC (1.27 ± 0.17) groups compared to that in the UV-Con and UV-Positive groups. The UV-PC treatment alleviated the clinical symptoms compared to the UV-Con group (*p* < 0.05; Figure 1C).

### 2.4. Histopathology of Skin in UVB-Exposed Mice

**Figure 2:**
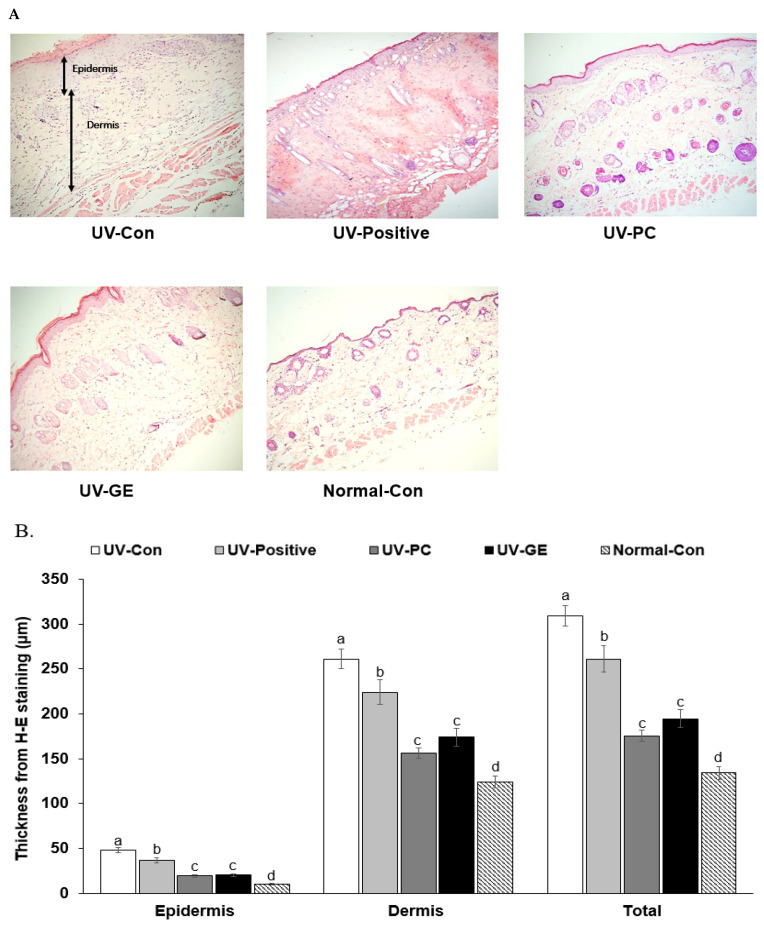

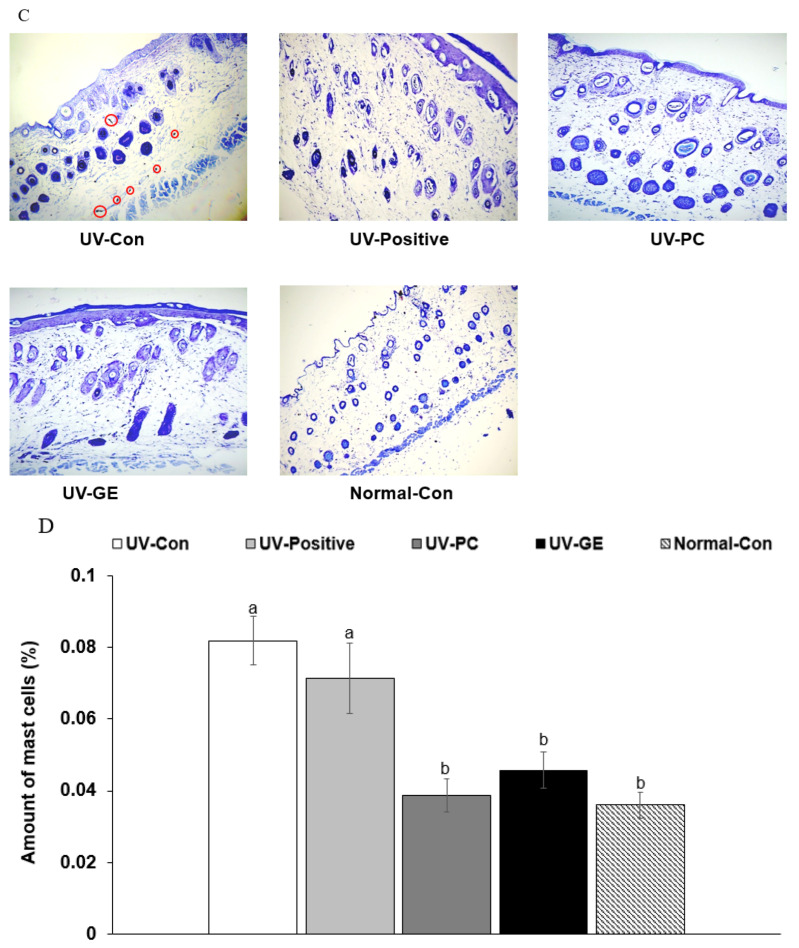
Histological differences and the number of mast cells in the dorsal skin. After the 21-day treatment, the dorsal skin was fixed with 10% formaldehyde, embedded in paraffin, and sections were made. The skin sections were stained with hematoxylin-eosin and toluidine blue staining. The magnification of the image was 100×. (**A**) Histology of the dorsal skin by hematoxylin-eosin staining. (**B**) The thickness of the epidermis and dermis are indicated by black lines (unit: μm). (**C**) Mast cell staining by toluidine blue. (**D**) The number of mast cells is presented as black dots marked by red circles (% of the skin tissue area). ^a,b,c^^,d^ Means with different superscripts were significantly different among the groups in each parameter by the Tukey test at *p* < 0.05.

The pathological changes in the skin of UVB-irradiated mice (including keratinization, epidermal thickness, inflammatory cell penetration, and skin vascular deposition) are shown. In Figure 2A–D, skin thickness, the number of inflammatory cells, keratinization, and vascularization were higher in the UV-Con and UV-Positive groups than in the UV-PC and UV-GE groups. The thickness of the epidermis and dermis in the UV-Con and UV-Positive was also higher than that of the UV-PC and UV-GE groups (*p* < 0.05; Figure 2A,B). The thickness of the epidermis and dermis was higher in the UV-Con than UV-Positive groups, even though that in the UV-Positive group was higher than in the UV-PC and UV-GE groups (*p* < 0.05; Figure 2A,B). The total thickness of the epidermis and dermis decreased in the order of the UV-Con (310 ± 11.4), UV-positive (261 ± 14.7 μm), UV-GE (195 ± 10.4 μm), UV-PC (176 ± 6.24 μm), and Norma-Con (134 ± 7.33 μm; Figure 2B). The number of mast cells in the UV-Con group was higher in the UV-Con and UV-positive groups than the UV-PC and UV-GE groups (Figure 2C,D). The number of mast cells in the UV-PC and UV-GE groups was similar to the Normal-Con (*p* < 0.05; Figure 2C,D).

### 2.5. Histopathological Analysis of the Colon

**Figure 3:**
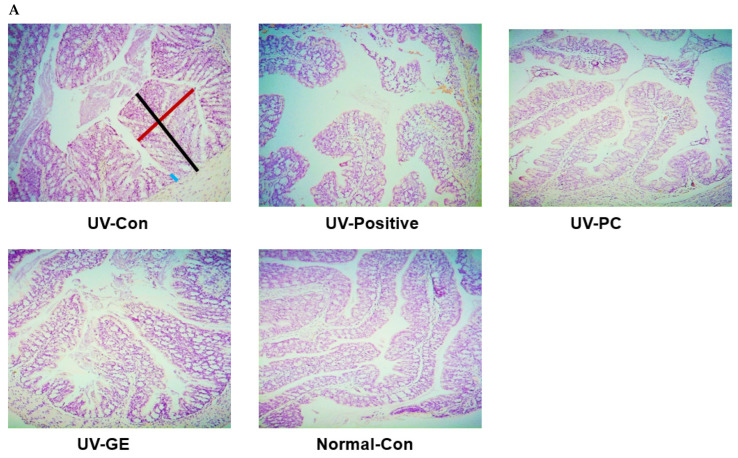

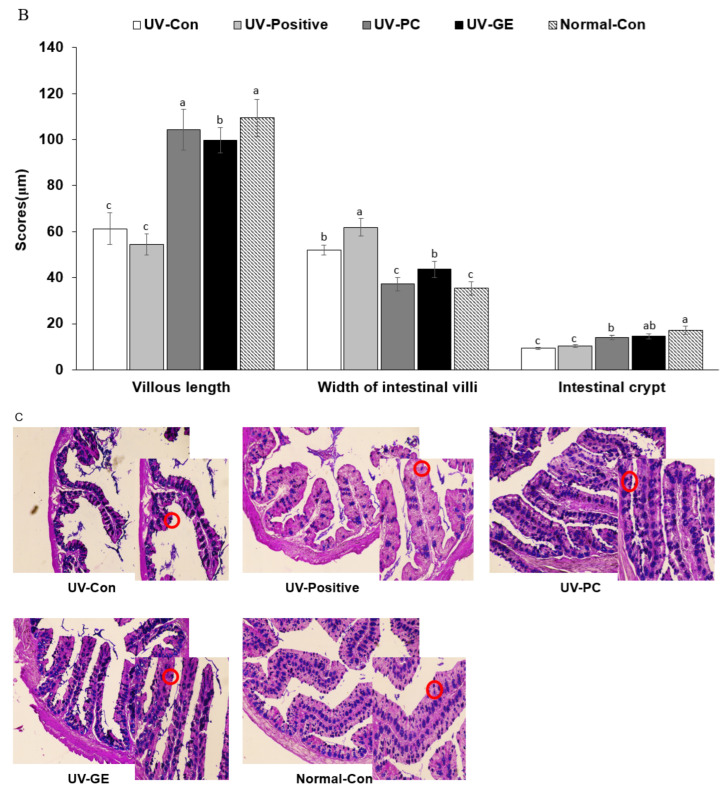

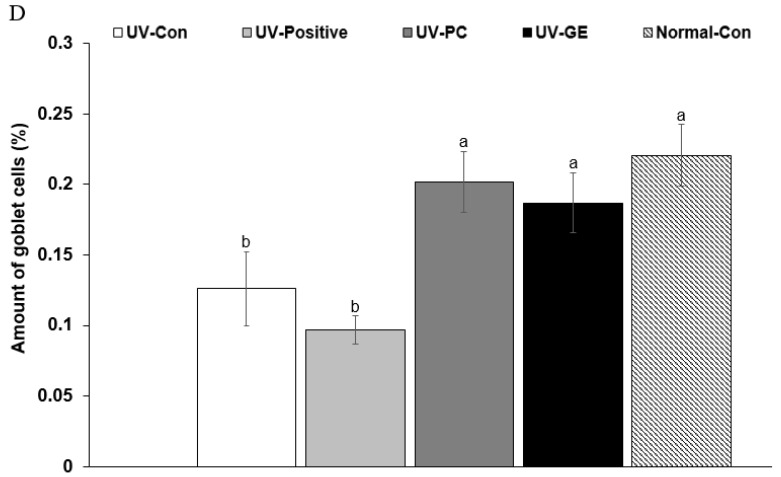
Histological differences and the amounts of mucin in the intestinal tissues among different groups. The intestinal tissues were fixed with 10% formaldehyde, embedded in paraffin, and sections were prepared. The large intestine sections were stained with hematoxylin-eosin and toluidine blue. The magnification of the image was 100×–200×. (**A**) Histology of the intestinal tissues by hematoxylin and eosin staining. Redline: Large intestine villi width, Blackline: Length of the large intestine villi, Blueline: Intestinal crypt. (**B**) Scores of the villi length, intestinal crypt, and large intestine villi width(mm). The higher scores (%), the healthier the colon. (**C**) Percentage of mucin in the large intestinal tissue Goblet cells producing mucin were marked with red circles in the large intestinal tissues. (**D**) Intestinal goblet cell score (%). ^a,b,c^ Means with different superscripts were significantly different among the groups in each parameter by the Tukey test at *p* < 0.05.

The villi of the large intestines of the UV-Con and UV-positive groups were shorter than the other groups. Shortening the intestinal villi was prevented in the UV-PC and UV-GE groups, while their length was extended in the UV-PC, similar to the Normal-Con (*p* < 0.05; Figure 3A,B). By contrast, the villi width was enlarged in the UV-Con and UV-Positive more than the Normal-Con, while UV-PC and UV-GE prevented the expansion of the width. In the UV-Con group, the intestinal crypt was shallower in the UV-Con than the Normal-Con, and it was enhanced with UV-GE and UV-PC (Figure 3A,B). The UV-Con and UV-Positive groups had shorter and broader villi and shallower crypt than others, indicating some inflammation in the intestines (*p* < 0.05; Figure 3A,B).

Mucin adheres to the mucosal epithelium of the large intestines, and mucin was stained blue by AB-PAS staining (Figure 3C). The mucin content was lowest in the UV-Con (0.12 ± 0.03%) and UV-Positive (0.10 ± 0.01%) groups (*p* < 0.05; Figure 3C,D). After the treatment with UV-PC and UV-GE, reducing the mucin amounts in the large intestine in UVB-exposed mice was prevented (UV-PC, 0.20 ± 0.02%; UV-GE, 0.19 ± 0.02%; Normal-Con, 0.22 ± 0.02%; *p* < 0.05; Figure 3C,D).

### 2.6. Skin and Liver Damage Index

The malondialdehyde contents as lipid peroxides in the back-skin tissues were higher in the UV-Con (28.51 ± 3.84) than the Normal-Con (16.40 ± 2.80) and similar between UV-Con and UV-Positive (31.73 ± 2.48). Its contents in the UV-PC (18.98 ± 1.71) and UV-PG (14.37 ± 1.49 nmol/mg protein) groups decreased as much as in the Normal-Con group (Table 2). The lipid peroxide contents in the liver showed a similar trend in skin tissue, and the hepatic lipid peroxide contents were lower in the UV-PC and UV-PE groups than in the UV-Con group (Table 2). The serum AST concentrations were similar in the UV-Con and Normal-Con groups but increased in the UV-Positive group compared to the UV-Con (*p* < 0.05; Table 2).

The serum ALT concentrations were similar in the UV-Con, UV-Positive, and Normal-Con groups (Table 2). The concentrations were lower in the UV-PC and UV-PE groups than in the UV-Con group (*p* < 0.05; Table 2). UVB irradiation itself induces somewhat liver damage, but the retinoic acid treatment in UV-Positive exacerbated liver damage. PC and GE did not damage liver function and improved it somewhat.

### 2.7. mRNA Expression Levels of Proinflammatory Cytokines in the Dorsal Skin

The serum TNF-α concentrations were higher in the UV-Con group than in the Normal-Con group, while the UV-PC and UV-GE groups showed protection against the increased TNF-α levels in UVB-exposed mice (*p* < 0.05; Table 2), indicating that UVB exposure increased the systemic inflammation and PC and GE prevented the increase.

**Figure 4:**
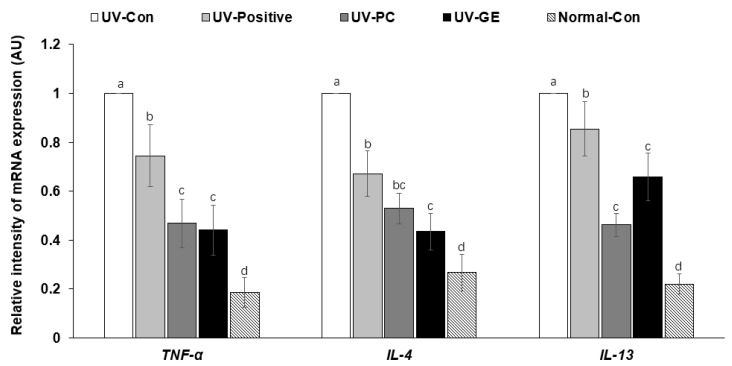
mRNA expression levels of proinflammatory cytokines in the dorsal skin. Each value represents the mean ± SD of 5 mice randomly selected from each group. AU, arbitrary unit. ^a,b,c^^,d^ Means with different superscripts were significantly different among the groups in each parameter by the Tukey test at *p* < 0.05.

The relative intensity of *TNF-α* mRNA expression in the back skin was much higher in the UV-Con group than in the Normal-Con group and decreased in the order of UV-Con (1 ± 0), UV-Positive (0.74 ± 0.14), UV-PC (0.47 ± 0.11), UV-GE (0.44 ± 0.10), and Normal-Con (0.19 ± 0.27; *p* < 0.05; Figure 4). *IL-4* and *IL-13* mRNA expression showed a similar tendency as TNF-*α* mRNA expression (*p* < 0.05; Figure 4). UV-GE decreased *IL-4* mRNA expression similar to the Normal-Con (Figure 4).

### 2.8. Short-Chain Fatty Acid Concentrations of the Blood from the Portal Vein

The serum acetate and propionate concentrations were similar in the UV-Con and Normal-Con groups, but the serum propionate concentration in the UV-PC group was higher compared to that in the UV-Con group (*p* < 0.05; Table 3). The serum butyrate concentrations were similar in the UV-Con (0.184 ± 0.002), UV-GE (0.202 ± 0.006 mM), and UV-Positive (0.200 ± 0.008 mM) groups. On the other hand, the UV-PC group (0.208 ± 0.006 mM) showed increases in serum butyrate concentrations similar to Normal-Con (0.211 ± 0.012 mM; *p* < 0.05; Table 3).

### 2.9. Gut Microbiota Community

**Figure 5:**
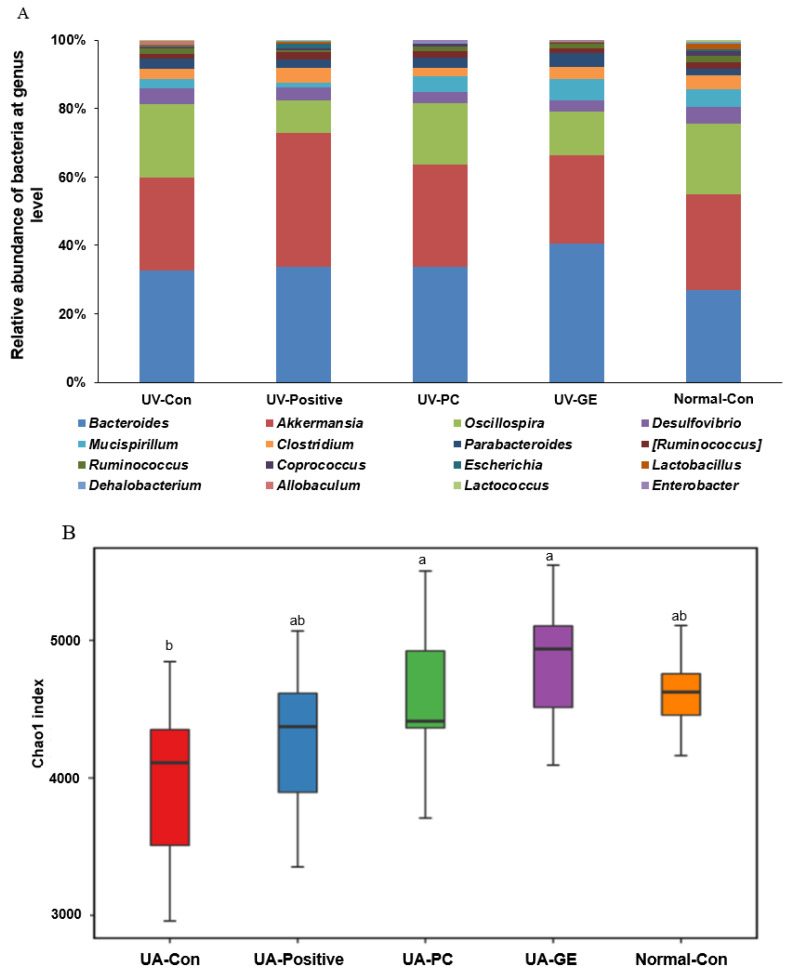

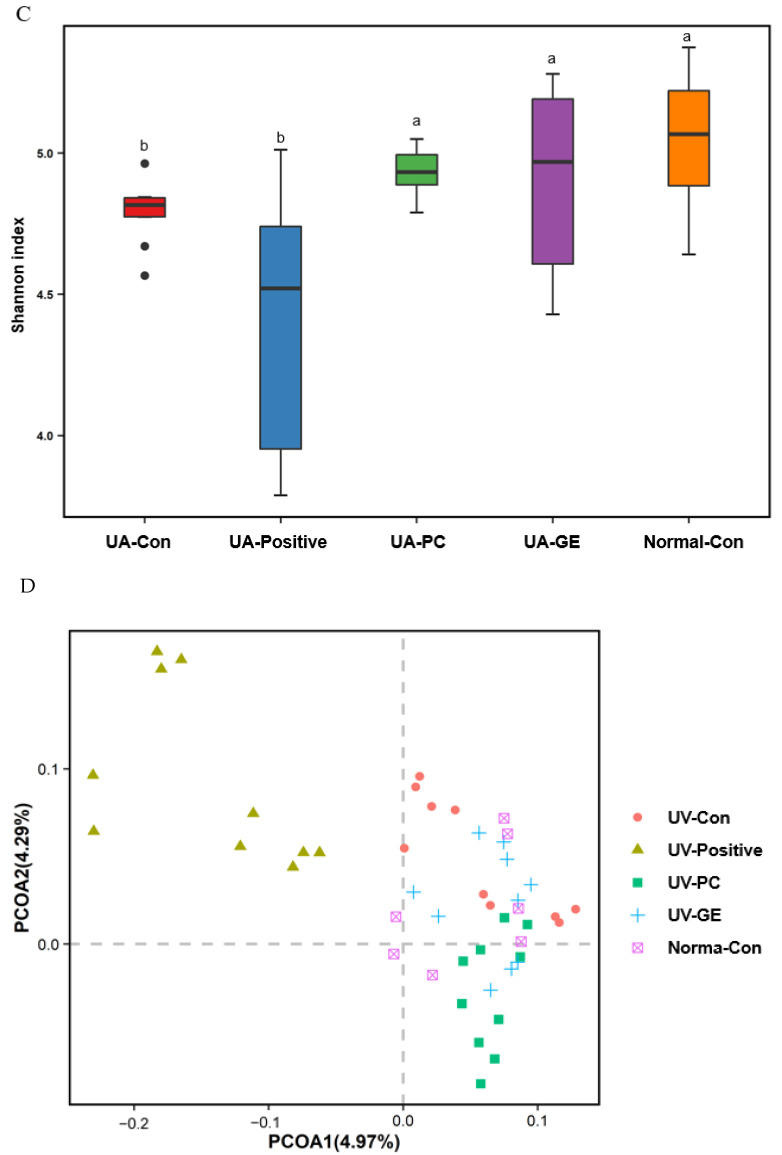

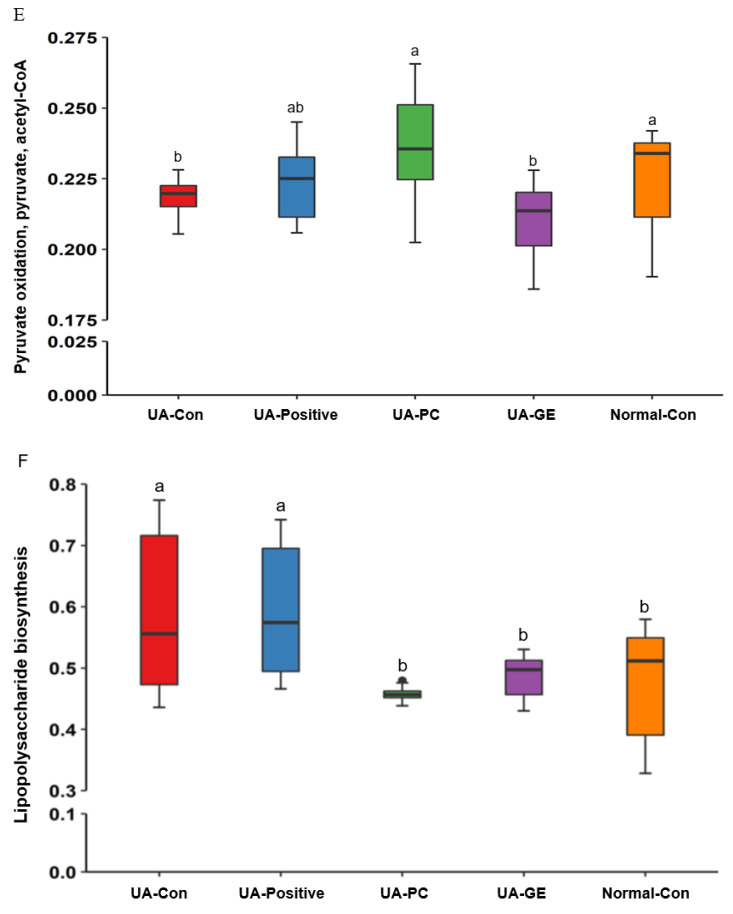

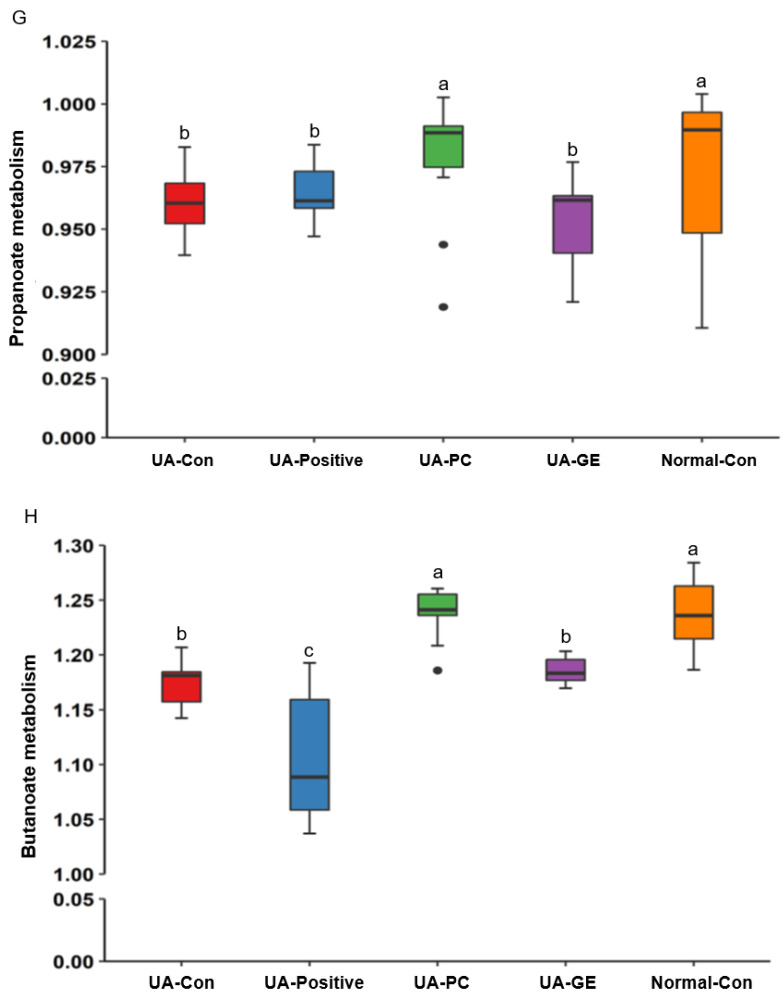
The profiles of gut microbiomes. After 21-day treatment, feces from the cecum were collected, and their bacterial DNA was analyzed by the next-generation sequencing method. (**A**) The relative amount (%) of fecal bacteria in the genus level. (**B**) Chao1 index. (**C**) Shannon index. (**D**) The principal coordinate analysis (PCoA) of fecal bacteria. (**E**) Pyruvate oxidation, pyruvate, acetyl-CoA metabolism in fecal bacteria using Picrust2. (**F**) Lipopolysaccharide biosynthesis in fecal bacteria using Picrust2. (**G**) Propanoate metabolism in fecal bacteria using Picrust2. (**H**) Butanoate metabolism in fecal bacteria using Picrust2. Each value represents the mean ± SD of 10 mice in each group. ^a,b,c^ Means with different superscripts were significantly different among the groups in each parameter by the Tukey test at *p* < 0.05.

The α-diversity was assessed using the Chao1 and Shannon indices. The chao1 index was higher in the UV-PC and UV-GE groups than in the UV-Con group (*p* < 0.05; Figure 5B). The Shannon index was lower in the UV-GE and UV-Positive groups than in the UV-Con group (*p* < 0.05; Figure 5B). PCoA analysis was conducted to compare the fecal bacterial species to clusters based on the weighted UniFrac distances. The bacteria in UV-Con separated from those in the other groups at *p* < 0.001 (Figure 5C). Therefore, the differences between UV-Positive, UV-PC, and UV-GE may be related to the protection of skin lesions.

According to Picrust2 analysis, the functions of the genes in fecal bacteria were evaluated. The activities of pyruvate oxidation, pyruvate, and acetyl-CoA biosynthesis pathways in the UV-PC group were higher than in the UV-Con group. They were similar to those of the Normal-Con group (*p* < 0.05; Figure 5D). The same results were noted in propanoate metabolism analysis (*p* < 0.05; Figure 5D). The butanoate metabolism was significantly lower in the UV-GE and UV-Positive groups than in the UV-Con group (*p* < 0.05).

## 3. Discussion

The skin is the largest organ and one of the most complex organs in the human body [19]. Multiple weeks of UVB radiation cause photoaging, and acute and intense UVB radiation induces sunburn and photocarcinogenesis [20]. Recent studies have confirmed that UVB irradiation induces intestinal ecological imbalances through the gut microbiome-skin axis [21], which influences the imbalances in immunity to exacerbate UVB-related skin damage [22]. UV-induced skin damage is mainly protected against sunscreen, and its commonly used drugs are topical retinoids, 5-fluorouracil, and cosmeceuticals, including antioxidants, soy, tea, and ginseng [23]. UVB-induced damage is mainly linked to increased ROS and proinflammatory cytokines, and many traditional Chinese medicines have the functions of ROS elimination, anti-oxidation, and immune function regulation [24]. Based on these studies, we applied the PC and GE water extracts to the back skin and oral administration before UVB irradiation to demonstrate the prevention and alleviation of UVB-induced skin damage in an animal model with UVB irradiation, as shown in the previous studies [25,26]. PC and GE improved the intestinal bacteria community in UVB-exposed mice. Both PC and GE extracts alleviated the clinical symptoms by reducing the proinflammatory cytokines in the mice topically exposed to UVB.

PC includes indigestible polysaccharides and triterpenoids having anti-inflammatory, immunomodulatory, anti-cancer, and anti-hyperglycemic properties [17]. GE contains 4-hydroxybenzaldehyde, gastrodin, gastrol, gastrodigenin, and indigestible polysaccharides, and it has anti-inflammatory, antioxidant, memory-improving, and anti-aging properties to treat brain-related symptoms such as headache, dizziness, and epilepsy [27]. The primary components of both PC and GE are polyphenols and polysaccharides, and they act as active components to improve anti-inflammation, immunomodulation, and anti-oxidative stress [28].

Retinoic acid treatment as the Positive-control improves the UVB-induced clinical symptoms on the dorsal skin as much as the UV-PC group. However, the lipid peroxides and proinflammatory cytokines were higher in the dorsal skin than in the UV-Con, and the intestinal morphology was similar to the UV-Con. Furthermore, it caused adverse effects of liver damage, and as a result, it induced a dramatic, below-normal weight loss. The results suggested that retinoic acid seemed to improve the skin appearance and morphology but did not suppress oxidative stress and inflammation nor enhance the gut microbiome-skin axis in the present study. Other studies have also demonstrated that retinoic acid treatment induces dry skin, cheilitis, muscle and joint pain, dyslipidemia, headache, and hypercalcemia as side effects [29]. Furthermore, retinoic acid increases the parathyroid hormone, adversely affecting vitamin D metabolism [29]. Therefore, retinoid acid is not an optimal therapeutic agent for UVB-induced skin damage.

Although optimal UVB exposure increases vitamin D and nitric oxide production in the skin, reducing liver inflammation [30], UVB irradiation in the current study developed skin damage, consistent with a previous study [31] showing that erythema, burns, erosion, and wounds appeared in the skin of mice exposed to UVB light. UVB irradiation is the leading cause of accelerated skin damage by increasing oxidative stress and inflammation [31]. Previous studies have shown that UVB exposure triggers damage by inflammation in the kidney and liver and weakens the innate immune system, and UVB exposure is linked to the increase in proinflammatory cytokines and lipid peroxide contents in the dorsal skin [32,33,34]. The present study also demonstrated that UVB exposure damaged the skin and liver tissues by increasing inflammation, oxidative stress, and overactivated immunity by elevating TNF-a in the bloodstream. Therefore, the treatment should enhance not only skin damage but also liver damage. In the present study, UV-PC and UV-GE alleviated UVB-induced damage in the dorsal skin and liver. It indicated that the skin damage caused by UVB exposure damages the liver tissues, and PC and GE could be potential therapeutic agents for UVB -induced damage.

In previous studies, acute UVB irradiation deteriorates skin morphology to thicken the epidermis and dermis [31]. The present study also showed similar results on skin morphological changes and increased inflammation by UVB irradiation. The UV-Positive group showed improved thickening of the epidermis and dermis in mice but had no inhibitory effect on mast cells and eosinophils. Therefore, the clinical skin symptoms were better in the UV-Positive than UV-Con, but the inflammation state did not differ between the two groups. However, the UV-PC and UV-GE groups effectively reduced the thickness of the epidermis and dermis, the number of skin mast cells and eosinophils, and the morphological changes on the skin surface were alleviated.

The morphological changes under UVB irradiation are related to inflammation and vasodilation [31], which clinically manifests as sunburn. UVB radiation activates the transcription factor, NF-κB, leading to increases in proinflammatory cytokines, including TNF-α, IL-13, and IL-4 [19], similar to the results in the present study. Proinflammatory cytokines and lipid peroxides generated from the skin can go into the circulation to induce liver damage bidirectionally [35], as shown in the present study. An increase in TNF-α in the circulation and the *IL-13*, *IL-4*, and *TNF-α* expression levels was seen in the skin tissue in the UV-Con and UV-Positive mice in the present study. Unlike serum concentrations, UV-Positive decreased the mRNA expression of *TNF-α, IL-13*, and *IL-4* in the dorsal skin. Moreover, the UV-PC and UV-GE groups reduced their expression levels in the Normal-Con group. These results suggested that UV-PC and UV-GE treatments inhibited UVB-induced skin damage by enhancing anti-inflammatory and antioxidant activities and inhibiting foreign mediators in the back cells of UVB-irradiated mice. PC and GE intake have been reported to reduce inflammation in inflammatory diseases, such as arthritis, diabetes, neuronal disorders, and aging [36,37,38,39], suggesting that improving systemic inflammation may contribute to skin inflammation. Therefore, UVB exposure induces skin lesions by increasing the proinflammatory cytokines, and PC and GE protect against the skin lesions by reducing systemic and skin inflammation.

The research direction has turned to the research on intestinal microbes and short-chain fatty acids to decrease systemic inflammation and alleviate skin damage by UVB irradiation through the gut-skin axis [40,41,42]. The skin is a tight chemical and immunological barrier protecting against pathogens, xenobiotics, and water loss [43]. The drug is delivered with stratum corneum and tight junctions, but water-soluble components with high molecular weight are difficult to absorb to treat skin damage [43]. In herbal extracts, water-soluble and oligomer components are difficult to absorb [44]. Therefore, herbal extract topical treatments just provide moisture not to exacerbate the skin lesion. Their intake changes the systemic inflammation through the gut microbiome, and skin damage can be alleviated through the gut-skin axis.

Furthermore, the skin damage caused by UVB exposure modified intestinal morphology, including villi length, crypt size, and goblet cells in the present study. The results revealed skin damage by inflammation influences intestines similar to the liver. Previous studies have reported that the mucin barrier balance plays an essential role in the mutual symbiosis between the intestinal commensal bacteria and the host, and the regulation of mucin is an essential factor in the intestinal ecological balance [45]. In addition, the villi length and width in the large intestine correlate with the digestion and absorption of food. In contrast, the height of the villi and the depth of the intestinal crypts are indicators of intestinal function. In this study, the UV-Positive mice had shorter and wider intestinal villi with minimal mucin, similar to UV-Con, suggesting that the integrity of the intestinal epithelium was disrupted in UV-Con and UV-Positive [46]. PC and GE increased villus length and mucin content, maintaining the mucin barrier balance and gut health. There is an intrinsic link between intestinal morphology and skin damage [5].

The outer mucosal layer in the colon is enriched in mucin-degrading bacteria, including *Bacteroides acidifaciens* in mice and *Bacteroides fragilis*, Bifidobacteriaceae, and *Akkermansia muciniphila* in mice and humans [47]. The reduction of the mucin layer promotes mucus secretion to increase the thickness of the mucus layer [47]. On the other hand, increased inflammation overgrowth of *Akkermansia* is induced in type 2 diabetes, dextran sodium sulfate-induced colitis, and colorectal cancer [48], although it remains controversial. Interestingly, *Akkermentia* in the fecal bacteria was elevated in the UV-positive group than in the other groups. *Akkermentia* removes mucin, which can be normally produced to make a new mucin layer when removed by *Akkermentia* to act as beneficial bacteria in the gut [47]. Therefore, the overgrowth of *Akkermentia* may have harmful effects when the host cannot make sufficient mucin.

The α-diversity index (Chao1, Shannon index) has been reported to be associated with gut health and affects host immunity [49]. UVB exposure (UV-Con) decreased the α-diversity indices representing the gut microbial community richness and community diversity. It suggested that UVB irradiation modulated the gut microbiota-gut-skin axis. The α-diversity was significantly higher in the UV-PC and UV-GE groups than in the UV-Con group. B-diversity determined by PCoA cluster analysis showed differences in the composition of intestinal bacteria among the groups in the present study. In metagenome analysis determined by Picrust2, the UV-PC group showed higher pyruvate oxidation, pyruvate, acetyl-CoA, and propanoate metabolism than the other groups. SCFA are the primary energy source for intestinal cells [50] and act as the host gut microbiota communicator. Acetic, propionic, and butyric acids comprise 83% of the human gut SCFA, and propionic and butyric acids improve the host metabolism, including skin [51]. UVB-irradiated mice have reduced serum propionate and butyrate concentrations compared to the Normal-con. Only UV-PC significantly increased the serum propionate and butyrate concentration compared to the UV-Con group. This experiment showed that PC strengthened the intestinal barrier and promoted intestinal epithelial cell movement and gut microbiota, contributing to better alleviating UVB-induced skin damage through the gut microbiota-gut-skin axis.

The present study clearly demonstrated that skin inflammation and oxidative stress by acute UVB irradiation induced liver and intestinal damage through elevating proinflammatory cytokines, lipid peroxides, and SCFA concentrations in the circulation. Furthermore, it was linked to gut microbiota alteration. It suggested that skin damage by UVB exposure was involved in modulating the gut microbiota–gut–skin axis. The present study had some limitations. First, all mice had a 43 En% diet to influence gut microbiota to exacerbate systemic inflammation. However, all treatments with a low-fat diet have not been included, and it may show different effects of PC and GE treatment on skin damage by UVB exposure. Second, the PC and GE were treated not only orally but topically, suggesting that the improvement of skin lesions was not directly shown through the skin–gut–gut microbiota axis. However, the topical application was mainly provided with moisture daily at a low dosage. The primary components, especially in PC were polysaccharides, and they were difficult to be absorbed in the dorsal skin, although PC showed better improvement in skin damage than GF by modulating gut microbiota. It suggested that the skin–gut–gut microbiota axis would critically impact the PC and GE therapy of UVB-induced skin damage.

In conclusion, UVB induces skin lesions by increasing inflammation, and its exacerbation is linked to the gut microbiota, known as the skin–gut–gut microbiota axis. Topical application and oral intake (0.3 g/kg bw) of PC and GE alleviated the clinical symptoms of the skin lesions under intense UVB irradiation by reducing inflammation and oxidative stress and improving intestinal morphology. PC additionally improved the fecal bacteria related to energy metabolism, propionate metabolism, and butyrate metabolism better than other groups. PC produced better improvement of the skin lesion than GE. Hence, PC reduced UVB-induced skin lesions by reducing the oxidative stress and inflammation potentially linked to the skin–gut microbiota axis.

## 4. Materials and Methods

### 4.1. Extraction, Lyophilization, and Quantification of Phenolics

GE and PC were purchased from an herbal market on 20 September 2019, and Dr. Young Seong Joo (Woosuk University, Jeonju, Korea) confirmed the authenticity. GE and PC were stored as voucher specimens, HU-H92 and HU-H93, respectively. Each herb was extracted in water (1:10) for 2 h at 95 °C and centrifuged at 8000× *g* for 30 min. The supernatants were separated, and the residues were reextracted in water (1:10) for 2 h at 95 °C. It was centrifuged, and the supernatants were separated and mixed with the previous one. Total supernatants were concentrated with vacuum evaporate and then lyophilized in a freeze-drier (Il Shin, Dongdochun-Si, Korea). The yields of GE and PC were 26.5 and 28.7%, respectively. Lyophilized GE and PC were dissolved in 1,3-butanediol (BG) at a final concentration of 0.1% and filtered. Retinoic acid was dissolved into the BG at 0.007% and filtered. The BG solution with herbal extracts was used as a local application to the dorsal skin. BG without extracts was applied to the dorsal skin of the mice in the control and normal-control groups.

The total polyphenol contents were determined using the Folin–Ciocalteu method [52]. After 3 min, 10% (*w*/*v*) Na_2_CO_3_ was added to each reaction mixture of each herbal extract. The reactions were performed in the dark for 60 min, and absorbances at 725 nm were recorded using a UV spectrophotometer (JASCO Inc., Tokyo, Japan). The total flavonoid contents were measured using a slight modification of the methods designed by Sulaiman et al. [53]. Each extract was mixed with 5% sodium nitrite. After 5 min, 10% aluminum chloride (3:1:1, *v*/*v*/*v*; Sigma, St. Louise, MO, USA) was added. Each mixture was neutralized with 1N NaOH after incubating it at room temperature for 6 min, and its optical density was measured at 510 nm using a UV spectrophotometer (Perkin Elmer, Boston, MA, USA). The total phenol and flavonoid contents were quantified with a standard curve made with 0.05, 0.1, 0.25, 0.5, 0.75, and 1 mg/mL levels of standard gallic acid and quercetin (Sigma, St. Louise, MO, USA), respectively. They are expressed as mg gallic acid equivalents (GAE) and quercetin equivalent (QE) per gram.

### 4.2. Animal Care

Fifty-seven-week-old male ICR mice (weighing 28–35 g) were purchased from Daehan Biolink (Eumsung, Korea). The mice were raised in a temperature, humidity, and ventilation-controlled environment at 23 °C, with a 12 h light/dark cycle. They consumed regular feed and sterilized distilled water for the one-week adaptation period. This study was approved by the Animal Care and Use Committee of Invivo company, Korea (IV-RB-02-2106-18) and was conducted according to the NIH Guide for the Care and Use of Laboratory Animals guidelines.

### 4.3. Experimental Design, UV-B Exposure, and Herbal Treatments

**Figure 6:**
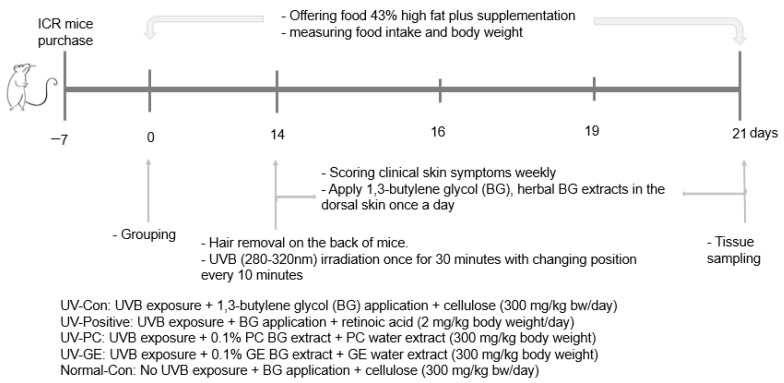
Experimental design.

To determine the local application and intake effects of PC and GE, mice recived UV-B exposure in the UV-Con, Positive-control, and experimental groups (UV-PC and UV-GE), while those in the normal-control were not exposed to UV-B. Our preliminary study determined the dosages of GE and PC in a cell-based study. Their contents in 5–20 ug/mL showed preventive effects on cell death by UV-B exposure in HaCaT cells. When it was calculated to animal dosage, it was about 0.3 g/body weight for animals and 200 mg/day for humans. Figure 6 presents the experimental design. The mice were divided and named into five groups: (1) UV exposure + 1,3-butanediol (BG) application into dorsal skin + cellulose intake (UV-Con; control group); (2) UV exposure + retinoic acid application into the dorsal skin + its intake (UV-Positive; Positive-control group); (3) UV exposure + PC BG solution application into the dorsal skin + its lyophilized water extract intake (UV-PC); (4) UV exposure + GE BG solution application into the dorsal skin + its lyophilized water extract intake (UV-GE); and (5) no UV exposure + BG application into the dorsal skin + cellulose intake (Normal-Con). The lyophilized PC and GE water extracts (0.3 g/kg body weight/day) were orally provided by a feeding needle to the UV-PC and UV-GE mice for two weeks, while retinoic acid (2 mg/kg body weight/day; TCI Chemical, Japan) was fed to the positive-control mice. Retinoic acid is reported to require retinoic acid to repair the epidermis after acute UVB irradiation [54,55,56], and it was used as a positive-control. The control and normal-control mice had oral cellulose feeding (0.3 g/kg body weight/day) since PC and GE water extracts mainly contained dietary fibers. In the third week, the back hair was removed after anesthesia with a mixture of ketamine and xylazine (100 and 10 mg/kg body weight), and the next day, BG solution of 0.1% PC extract, 0.1% GE extract (200 µL), or 0.007% retinoic acid was applied to the dorsal skin. 1,3-butylene glycol (200 µL) was applied to the dorsal skin in the control and Normal-control groups. After 30 min, to allow complete absorption of BG solution in the skin [25], the mice were anesthetized and exposed to 100 mJ/cm^2^ UVB intensity (280–320 nm) for 30 min in a closed chamber (Spectroline TM XL-1000, Spectronics Corp., Westbury, NY, USA) where the UVB light was placed 30 cm above the mice [3]. The positions of the mice were switched every 10 min (three times in total) during UVB exposure. The normal-control mice were laid down under fluorescent light (20W) in the room instead of UVB after being anesthetized [57]. The mice had oral intake and local application on the dorsal skin by the assigned treatment for one week after UVB exposure.

All mice were given a high-fat diet containing 17 energy% (En%) protein (casein and methionine), 40 En% carbohydrates (corn starch and sugar), and 43 En% fat (corn oil and shortening) contents. Cellulose, minerals, vitamins, and choline were added to the diet to meet the American Institute of Nutrition for rodents [58]. The diet composition of the high-fat diet was similar to the nutrient requirement for maintaining adult rats [59], which people in Western countries consume [60]. Therefore, a high-fat diet was freely provided in the present study. The dietary intake and body weight were recorded weekly during the experimental periods. Energy efficiency was calculated according to the equation by dividing the energy intake by body weight gain after UV irradiation. When the mice had pain or inflammation, energy efficiency was lower.

### 4.4. Evaluation of Clinical Symptoms by Acute UVB Irradiation

The clinical manifestations of erythema, wound, burn, and erosion after UV light induction were induced. The dorsal skin of the mice was evaluated at zero, two, five, and seven days after UV irradiation (erythema, burns, erosions, wound). A visual assessment of the clinical symptoms was used to evaluate the clinical skin damage symptoms (no symptom: 0, mild: 1, moderate: 2, severe: 3) [26,61].

### 4.5. Sample Collection and Tissue Preparation

At the end of the experimental period, blood was collected from the inferior vena cava and portal vein, and serum was separated from centrifuging blood at 6000 rpm at 4 °C for five minutes using a micro high-speed centrifuge micro17TR (Hanil, Daejeon, South Korea). The liver, large intestine, visceral fat, and skin tissues were dissected and collected from the ICR mice, and the visceral fat mass was weighed with a precision balance (Sartorius, Göttingen, Germany). The feces in the cecum was also collected to measure bacterial community by next-generation sequencing (NGS). Serum concentrations of alanine aminotransferase (ALT) and aspartate aminotransferase (AST) were assessed using the ALT and AST kits from Asan Pharmaceutical (Seoul, Korea). The serum tumor necrosis factor (TNF)-α concentrations were measured using an enzyme-linked immunosorbent assay kit (BD Biosciences, Bedford, MA, USA).

The large intestine and skin tissues were stored in 10% formalin in 0.1% PBS for 24 h and dehydrated with xylazine and ethanol. The dehydrated tissues were embedded with paraffin and sectioned at 5 μm. The histology of the large intestines (villous length, intestinal crypt, the width of intestinal villi) and dorsal skin was evaluated in hematoxylin & eosin (H-E) staining. In brief, the section was stained with hematoxylin solution (Sigma, St. Loise, MO, USA) for the nucleus, washed with PBS, differentiated with 0.3% acid alcohol, washed with water, stained with eosin for the cytoplasm, and dehydrated, and mounted. The intestinal histology of the large intestines was determined with hematoxylin & eosin (H-E) staining to measure epidermis and dermis thickness, abnormality of cells and nucleus in the keratocytes, and skin surface. The number of goblet cells (%) on the intestinal villi was examined in Alcian Blue-Periodic acid (AB-Pas) staining. Toluidine blue (Sigma-Aldrich, St. Louis, MO, USA) staining was also performed to calculate the number of mast cells in the skin tissues (%). The above observations were magnified 100/200 times using an optical microscope (Axio Imager 2; Carl Zeiss AG, Oberkochen, Germany) and evaluated using I-Solution software, including a counter format to view histopathological changes.

### 4.6. Biochemical Test

The liver tissue was lysed in a methanol solution (1:10) with ultrasound and mixed with chloroform solution. After adding distilled water, the mixture was separated into two layers after centrifuging at 3000 rpm, at 4 °C, for 10 min. The upper layer containing the organic solvent was separated, and ethanol plus triton X mixture as emulsifiers (1:1 *v*/*v*) was added to the organic solvent part. The triglyceride contents in the emulsifier layer were measured using a triglyceride kit (Asan Pharmaceutical, Korea).

The liver or skin tissues were lysed in lysis buffer (1:10) with ultrasound. A mixture of cold 0.25N-HCl, 15% trichloroacetic acid, and 0.38% 2-thiobarbituric acid was added to the lysed liver tissue. The mixture was placed in a 100 °C incubator for two hours. After cooling, the supernatants were separated with centrifugation at 5000 rpm at 4 °C for 10 min, and their optical density was detected at 532 nm in a spectrophotometer (Perkin-Elmer, Boston, MA, USA). The contents of 2-thiobarbituric acid reactive substances (TBARs) were measured in the liver and skin tissue extract using TBARS assay kit (Abcam, Cambridge, UK).

The short-chain fatty acid (SCFA) contents of the serum from the portal vein were analyzed by gas chromatography (Clarus 680, PerkinElmer, Boston, MA, USA); 1 mM acetate, propionate, and butyrate (Sigma, St. Louise, MO, USA) were used as standards [62].

### 4.7. Skin Real-Time Quantitative PCR

Trizol (Ambion Inc., Austin, TX, USA) reagent solution was added to the skin tissue, and total RNA was isolated. The amount and purity of total RNA were determined using 260 nm and 280 nm in spectrometers (Perkin Elmer). According to the manufacturer’s manual, total RNA (1 µg) was synthesized into cDNA using the Superscript™ III Reverse Transcriptase Kit (Bio-Rad, Richmond, CA, USA). The synthesized cDNA was mixed with SYBR Green supermix (Bio-Rad, Richmond, CA, USA). Its amplification was performed using the CFX Connect™ Real-Time PCR Detection System (Bio-Rad Laboratories, Inc., Hercules, CA, USA). Relative mRNA expression of TNF-α, interleukin (IL)-4, and IL-13 in the skin was assessed using the method of Ct comparison, and the expression level of the gene was normalized to that of the housekeeping gene β-actin. Table S1 lists the primers for the genes of interest.

### 4.8. NGS of Gut Microbes

The total genomic DNA of fecal bacteria was extracted using a QIAamp Power Fecal DNA Kit (QIAGEN, 12830-50, Hilden, Germany). The gut microbiome in collected feces was determined using the NGS method, a high-throughput DNA sequencing technology. The extracted DNA samples were normalized to 5 ng/µL using DEPC Water (Sigma). The primers of the V3-V4 rRNA region, which contain sufficient genetic information on the bacteria, were used to amplify the fecal bacteria [63]. The extracted DNA, primers, and polymerase with proper buffers in the KAPA HiFi HotStart Ready Mix PCR Kit (KAPA Biosystems, Potters Bar, UK) were mixed and then amplified using GeneAmp PCR under the following conditions: 94 °C for three minutes, followed by 35 cycles: 94 °C for 15 s, 55 °C for 45 s followed by one minute at 72 °C, and eight min extension at 72 °C. The PCR amplicons were visualized on agarose gels using a QIAquick PCR purification kit (Qiagen, Valencia, CA, USA), and their concentrations were measured on a Nanodrop 2000 (ThermoFisher, Waltham, MA, USA). The amplified PCR products were purified using AMPure beads (Beckman Coulter, Brea, CA, USA), and the purified samples were sent to Macrogen Ltd. (Seoul, Korea) for sequencing using the NGS method.

The fasta data were analyzed using Mothur (version 1.43.0, Boston, MA, USA). The bidirectional sequences were first merged, and the sequences >200 bp were filtered. Clustering was performed, and the similarity threshold was 97%. The chimera was identified and removed with a default identification threshold of 3. The processed sequence files were annotated according to the Greengenes reference taxonomy. The α-diversity, Chao1, and Shannon indices were calculated using the “summary.single” tool with the bacteria counts and taxonomy files. Beta diversity was determined using an unweighted unifrac tool. Linear discriminant analysis (LDA) scores were calculated using the lefse command.

### 4.9. Metagenomic Function Analysis of Gut Microbiome by Picrust2 Analysis

Functional abundance analysis of the gene sequences was performed on the processed sequence (fasta) files and count files using Picrust2 (version 2.3.0_b, Boston, MA, USA). After the count file from the Mothur process was converted to the biom format, the sequence files and biom file were introduced into picrust2. The entire pipeline tool of picrust2 was run for metagenome analysis. The KEGG Orthology (KO) abundance was converted to the relative abundance, and the website (https://www.genome.jp/kegg/tool/map_pathway1.html, accessed on 6 January 2022) was used for alignment to obtain 341 related metabolic pathways.

### 4.10. Statistical Analysis

The results were analyzed by SPSS 16.0 (IBM SPSS, Armonk, NY, USA). The results are expressed as the mean and standard deviation (SD). One-way analysis of variance (ANOVA) was used between the groups, and multiple comparisons were conducted using Duncan’s test. The statistical significance was determined at *p* < 0.05.

## Figures and Tables

**Table 1:** Body composition and food intake at the end of experimental periods.

	UV-Con	UV-Positive	UV-PC	UV-GE	Normal-Con
Final weight (g)	33.6 ± 0.84 ^b^	25.6 ± 0.84 ^c^	32.2 ± 0.87 ^b^	36.6 ± 0.85 ^a^	36.0 ± 1.07 ^a^
Weight gain (g)	3.40 ± 0.82 ^b^	-1.35 ± 0.94 ^c^	3.10 ± 0.76 ^b^	4.97 ± 0.91 ^a^	4.84 ± 0.88 ^a^
Food intake (g/day)	4.66 ± 0.19	4.51 ± 0.22	4.55 ± 0.38	4.89 ± 0.25	4.16 ± 0.18
Food efficiency	0.73 ± 0.27 ^b^	−0.29 ± 0.21 ^c^	0.71 ± 0.28 ^b^	1.13 ± 0.24 ^a^	1.20 ± 0.27 ^a^
Epididymal fat (g)	0.86 ± 0.13 ^a^	0.38 ± 0.11 ^b^	0.82 ± 0.13 ^a^	1.02 ± 0.09 ^a^	0.88 ± 0.14 ^a^
Retroperitoneal fat (g)	0.38 ± 0.07 ^a^	0.13 ± 0.03 ^b^	0.31 ± 0.05 ^a^	0.41 ± 0.05 ^a^	0.43 ± 0.08 ^a^
Total visceral fat (g)	1.24 ± 0.18 ^a^	0.51 ± 0.14 ^b^	1.12 ± 0.17 ^a^	1.43 ± 0.14 ^a^	1.31 ± 0.22 ^a^

Values represented means ± standard deviations (n = 10). UV-Con, UVB exposure + 1,3-butylene glycol (BG) application + cellulose (300 mg/kg bw/day); Positive-control, UVB exposure + BG application + retinoic acid (2 mg/kg body weight/day); UV-PC, UVB exposure + 0.1% PC BG extract + PC water extract (300 mg/kg body weight); UV-GE, UVB exposure + 0.1% GE BG extract + GE water extract (300 mg/kg body weight); Normal-Con, No UVB exposure + BG application + cellulose (300 mg/kg bw/day). Food efficiency was obtained by dividing the weight gain by the food intake. Different letters indicated a significant difference (*p* < 0.05). ^a,b,c^ Different letters indicated a significant difference at *p* < 0.05.

**Table 2:** Skin and liver damage.

	UV-Con	PA–Positive	UV-PC	UV-GE	Normal-Con
Skin TBARs (nmol/mg protein)	28.51 ± 3.84 ^a^	31.73 ± 2.48 ^a^	18.98 ± 1.71 ^b^	14.37 ± 1.49 ^c^	16.40 ± 2.80 ^bc^
Liver TBARs (nmol/mg protein)	48.50 ± 2.40 ^a^	55.83 ± 2.65 ^a^	41.37 ± 2.74 ^bc^	38.15 ± 2.50 ^c^	43.03 ± 3.62 ^b^
Serum TNF-α (ng/mL)	13.2 ± 3.15 ^a^	12.4 ± 2.82 ^a^	7.25 ± 1.13 ^b^	6.16 ± 0.91 ^b^	5.18 ± 0.69 ^b^
Serum AST (mg/dL)	47.9 ± 1.87 ^b^	68.5 ± 1.28 ^a^	40.9 ± 3.00 ^b^	39.2 ± 1.21 ^b^	44.9 ± 3.26 ^b^
Serum ALT (mg/dL)	19.6 ± 0.57 ^a^	20.3 ± 1.69 ^a^	10.4 ± 1.31 ^bc^	6.09 ± 0.45 ^d^	14.8 ± 2.67 ^b^

Values represented means ± standard deviations (n = 10). UV-Con, UVB exposure + 1,3-butylene glycol (BG) application + cellulose (300 mg/kg bw/day); Positive-control, UVB exposure + BG application + retinoic acid (2 mg/kg body weight/day); UV-PC, UVB exposure + 0.1% PC BG extract + PC water extract (300 mg/kg body weight); UV-GE, UVB exposure + 0.1% GE BG extract + GE water extract (300 mg/kg body weight); Normal-Con, No UVB exposure + BG application + cellulose (300 mg/kg bw/day). TBARs, 2-thiobarbituric acid reactive substances; TNF-α, tumor necrosis factor-α; AST, aspartate aminotransferase; ALT, alanine aminotransferase. ^a,b,c,d^ Different letters indicated a significant difference at *p* < 0.05.

**Table 3:** Short-chain fatty acids (SCFA) concentration in serum from the portal vein.

	UV-Con	UV-Positive	UV-PC	UV-GE	Normal-Con
Acetic acid (mM)	0.217 ± 0.006	0.235 ± 0.011	0.254 ± 0.010	0.238 ± 0.012	0.242 ± 0.021
Propionic acid (mM)	0.147 ± 0.001 ^b^	0.159 ± 0.006 ^ab^	0.161 ± 0.003 ^a^	0.149 ± 0.002 ^ab^	0.152 ± 0.004 ^ab^
Butyrate acid (mM)	0.184 ± 0.002 ^b^	0.200 ± 0.008 ^ab^	0.208 ± 0.006 ^a^	0.202 ± 0.006 ^ab^	0.211 ± 0.012 ^a^
Total SCFA (mM)	0.440 ± 0.057 ^b^	0.543 ± 0.046 ^ab^	0.623 ± 0.014 ^a^	0.591 ± 0.013 ^a^	0.571 ± 0.054 ^a^

Values represented means ± standard deviations (n = 10). UV-Con, UVB exposure + 1,3-butylene glycol (BG) application + cellulose (300 mg/kg bw/day); Positive-control, UVB exposure + BG application + retinoic acid (2 mg/kg body weight/day); UV-PC, UVB exposure + 0.1% PC BG extract + PC water extract (300 mg/kg body weight); UV-GE, UVB exposure + 0.1% GE BG extract + GE water extract (300 mg/kg body weight); Normal-Con, No UVB exposure + BG application + cellulose (300 mg/kg bw/day). ^a,b^ Different letters indicated a significant difference at *p* < 0.05.

## Data Availability

The data will be available upon request to the corresponding author.

## Supplementary Materials

The following supporting information can be downloaded at: https://www.mdpi.com/article/10.3390/ijms231810833/s1, Table S1: Primers of real time PCR.

## Abbreviations

GE, *Gastrodia elata* Blume; PC, *Poria cocos*; UVB, ultraviolet B; ROS, reactive oxygen species; ERK, extracellular signal-regulated kinases; JNK, c-Jun N-terminal kinase; MAPK, mitogen-activated protein kinase; MMP, matrix metalloproteinase; BG, 1,3-butanediol; UV-Con, UVB exposure + BG application + cellulose intake; UV-Positive, UVB exposure + retinoic acid application + its intake; UV-PC, UVB exposure + *Poria cocos* BG extract application + its water extract intake; UV-GE, UVB exposure + *Gastrodia elata* Blume BG extract application + its water extract intake; Normal-control, no UVB exposure + BG application + cellulose; En%, energy%; ALT, alanine aminotransferase; AST, aspartate aminotransferase; NGS, next-generation sequencing; TNF, tumor necrosis factor; HE, hematoxylin–eosin; AB-PAS, alcian blue-periodic acid solution; TBAR, 2-thiobarbituric acid reactive substance; SCFA, short-chain fatty acid; IL, interleukin.
