# Supplementary material for: Beneficial Effect of Gastrodia elata Blume and Poria cocos Wolf Administration on Acute UVB Irradiation by Alleviating Inflammation through Promoting the Gut-Skin Axis
Source: Int J Mol Sci. 2022 Sep 16;23(18):10833. doi: 10.3390/ijms231810833 (PMC9504230; doi:10.3390/ijms231810833)
Supplement: Supplementary file 1 [file ijms-23-10833-s001.zip › ijms-1875915-supplementary.pdf]

Table S1. Primers of real time PCR

Gene	Quantification method	Sequence (5′-3′)	Tm (°C)
β-actin	Forward primer	5'- TCCTGTGGCATCCACGAAACT -3'	60.9
	Reverse primer	5'- GAAGCATTTGCGGTGGACGAT -3	60.6
TNF-α	Forward primer	5'- CCTGTAGCCCACGTCGTAGC -3'	56.9
	Reverse primer	5'- TTGACCTCAGCGCTGAGTTG -3'	56.1
IL-13	Forward primer	5'- CAGCTCCCTGGTTCTCTCAC -3'	53.4
	Reverse primer	5'- CCACACTCCATACCATGCTG -3'	53.3
IL-4	Forward primer	5'- GAATGTACCAGGAGCCATATC -3'	50.0
	Reverse primer	5'- CTCAGTACTACGAGTAATCCA -3	43.4
